# Differential responses in dorsal visual cortex to motion and disparity depth cues

**DOI:** 10.3389/fnhum.2013.00815

**Published:** 2013-12-02

**Authors:** David M. Arnoldussen, Jeroen Goossens, Albert V. van den Berg

**Affiliations:** Section Biophysics, Department of Cognitive Neuroscience, Donders Institute for Brain, Cognition, and Behavior, Radboud University Nijmegen Medical CentreNijmegen, Netherlands

**Keywords:** disparity, V6, motion, self-motion, optic flow, CSv, stereo

## Abstract

We investigated how interactions between monocular motion parallax and binocular cues to depth vary in human motion areas for wide-field visual motion stimuli (110 × 100°). We used fMRI with an extensive 2 × 3 × 2 factorial blocked design in which we combined two types of self-motion (translational motion and translational + rotational motion), with three categories of motion inflicted by the degree of noise (self-motion, distorted self-motion, and multiple object-motion), and two different view modes of the flow patterns (stereo and synoptic viewing). Interactions between disparity and motion category revealed distinct contributions to self- and object-motion processing in 3D. For cortical areas V6 and CSv, but not the anterior part of MT^+^ with bilateral visual responsiveness (MT^+^/b), we found a disparity-dependent effect of rotational flow and noise: When self-motion perception was degraded by adding rotational flow and moderate levels of noise, the BOLD responses were reduced compared with translational self-motion alone, but this reduction was cancelled by adding stereo information which also rescued the subject's self-motion percept. At high noise levels, when the self-motion percept gave way to a swarm of moving objects, the BOLD signal strongly increased compared to self-motion in areas MT^+^/b and V6, but only for stereo in the latter. BOLD response did not increase for either view mode in CSv. These different response patterns indicate different contributions of areas V6, MT^+^/b, and CSv to the processing of self-motion perception and the processing of multiple independent motions.

## Introduction

Depth can be perceived with a single eye when an object (or a whole scene) approaches. The visual flow of the approaching scene gives rise to relative motion between the images of nearer and farther elements of the scene: motion parallax.

The monocular depth information from motion parallax is normally extended with stereoscopic cues to depth. Recent fMRI studies reported differential processing of these cues in different visual cortical areas (Rokers et al., 2009; Ban et al., 2012; Seymour and Clifford, 2012). Depth cues from motion parallax and stereo interact most convincingly in area V3b/KO (Ban et al., 2012).

For natural movement through the world, depth analysis is often far more complicated. Firstly, independently moving elements violate the property that the motion-parallax cue to depth depends on a rigidly moving scene or object relative to the observer. Independently moving elements make relative motion ambiguous: it can be caused by object movement in the world, by depth or both.

Secondly, rotations of the eye add a large common motion to the retinal flow, which can completely mask relative motion due to depth (Koenderink and Doorn, 1987). Hence, self motion with a strong component of rotation provides poor depth information compared to pure translational self motion. Here, we investigate whether these different complications reveal specific types of interaction between monocular and binocular cues to depth.

We distinguish two main functions for depth cue combination:

1. Identification of the direction of self-motion in depth (Lappe et al., 1999; Britten, 2008). When the retinal flow simulates forward motion of the eye through the world (self-motion), degradation of the flow pattern's structure by noise reduces the percept of self-motion. Stereo information is known to improve the heading percept in this case by revealing the depth order of the scene (Van Den Berg and Brenner, 1994a,b). Hence we expect for a cortical region involved in self motion perception a change in the BOLD signal when noise is added to the flow, which then is countered by the addition of stereo signals.
2. The parsing of the visual flow into self-motion and independent object motion (Rushton et al., 2007; Warren and Rushton, 2009a), or the determination of the depth components of independently moving objects.

When one moves through a stationary scene, the flow reveals the self-motion. Deviations from the flow, however, reveal the objects that are also moving relative to the scene. This is called flow-parsing. Cortical areas involved in flow parsing may reveal a flow parsing component of the BOLD signal that grows when more objects move independently or, when each moving object's deviation from the flow becomes larger. Stereo signals may further characterize the deviation, leading to a steeper increase of the BOLD signal as a function of the deviation from the normal flow. One can follow the same reasoning for a stationary observer (zero flow). Then one arrives at the same predictions for the BOLD responses but now irrespective of objects' 3D motions relative to the self-motion pattern. Hence our prediction does not distinguish whether the pattern of BOLD interactions is specific to flow parsing or 3D motion of independently moving objects *per se*.

Here, we used wide-field optic flow stimuli with manipulation of the motion parallax cue to depth and looked for signs of stereo signals interacting with that manipulation.

We presented these stimuli using a custom-made MRI bore projection system that allowed for precise perspective projected wide-field optic flow to both eyes. This is important for the study of self-motion, because of the large receptive fields in self-motion sensitive areas (see supplemental information).

We first show, in a perceptual study that the introduction of noise evokes a transition of the percept: from strong self-motion (the scene moving as a whole relative to the eye), into weak self-motion, on to multiple objects moving independently without a self-motion percept. This allows us to classify our stimuli into different categories evoking a self-motion percept or not and observe when stereo signals make a difference.

To investigate interactions between depth cues that are relevant for self motion perception, we contrasted the BOLD responses to stimuli with different amounts of noise that still evoked a self-motion percept. Also, we contrasted BOLD responses to stimuli that evoked a percept of only independently moving objects with BOLD responses to stimuli with pure self-motion. These different comparisons revealed, within dorsal motion cortex, different interactions between stereo and motion cues to depth, indicating contributions of stereo to self-motion perception and flow parsing/object motion.

## Materials and methods

### Subjects

Twelve healthy subjects (1 left-handed) without stereo deficits participated in the fMRI experiment (age range: 23–47 years; 4 female). Five of the subjects also participated in the perceptual study. All subjects had normal or corrected-to-normal vision and had experience with optic flow stimuli, and previously participated in fMRI studies. Procedures were approved by the Radboud University Medical Centre, The Netherlands. All but one subject (one of the authors) were naïve to the purpose of the study. Informed consent was obtained in writing from all subjects prior to scanning. Subjects participated in two or three scanning sessions of ~120 min each.

### Visual stimuli

All visual stimuli were generated on a Macintosh MacBook-pro by using openGL-rendering software. For each eye, the scene consisted of a cloud of 500 dots (250 white and 250 black, point size: 1/3° diameter) on a gray background (luminances: white: 21.0 cd/m, black: 15 cd/m, gray: 2.0 cd/m). To simulate self-motion, dots moved within a simulated scene of (width × height × depth) 14 × 7 × 5 m in front of the head. Individual dots were drawn randomly and uniformly within the scene and had a lifetime of 2000 ms with asynchronous refresh times. Pixels had a size of 0.2–0.34°, depending on eccentricity (large at the center because of the nearer distance, smallest at largest eccentricity of 60°). Because we applied sub-pixel positioning by OpenGL software, displacement resolution of 1/10 th of the pixel size is offered, i.e., 0.02–0.034°.

**Figure 1:**
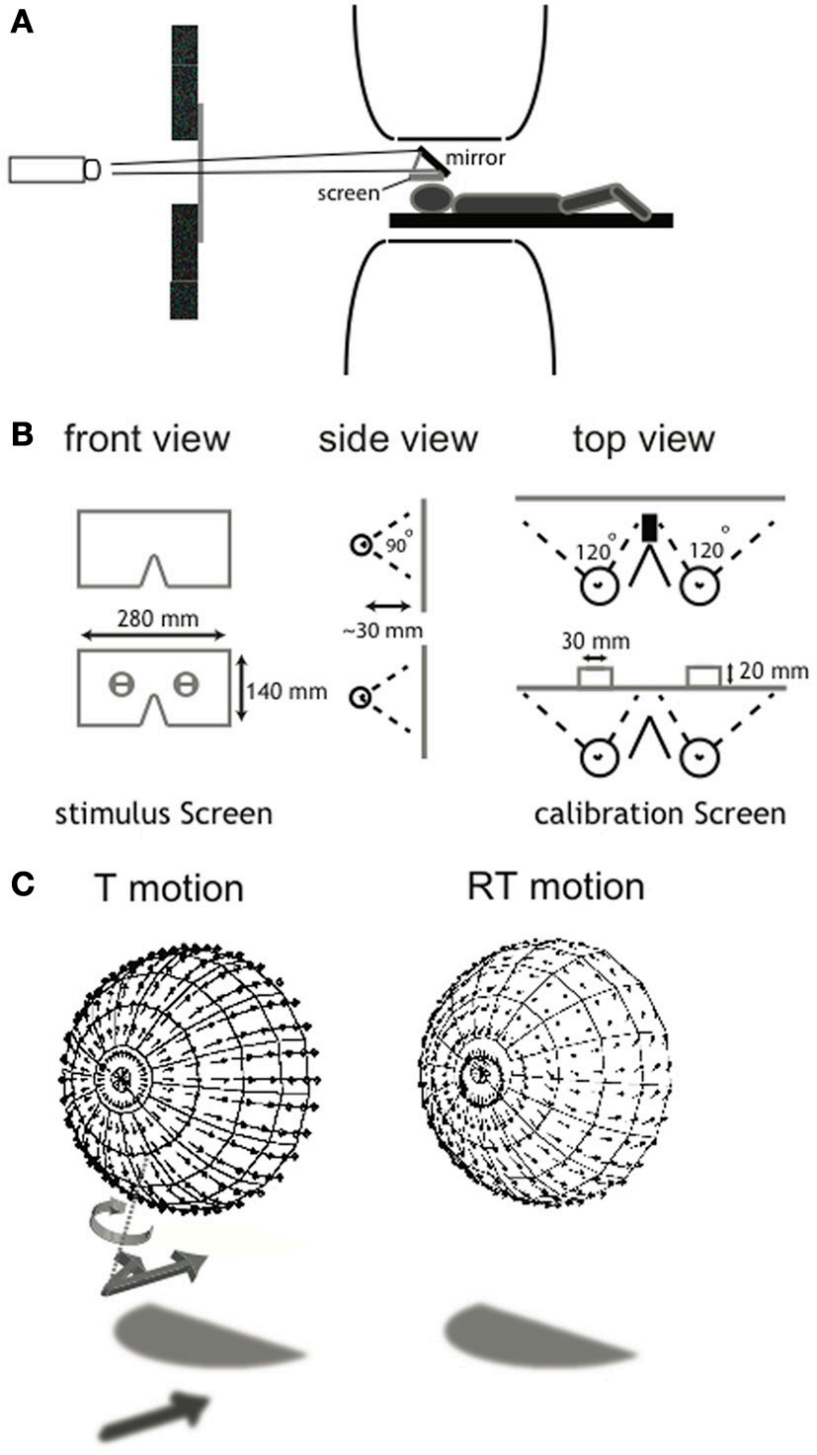
**Illustration of the wide-field visual projection set-up and the calibration procedure. (A)** A projector with a custom set of lenses projected a small projection over 4.5 m distance via a mirror on the projection screen (28 × 14 cm). Subjects wore contact lenses with high diffraction (>25 diopters) in both eyes and looked at a stimulus screen that was placed ~3 cm above the subject's eyes. The set-up allowed for a stimulus presentation with a field of view of ~110 × ~100°. **(B)** To assess the exact eye position relative to the screen, a calibration screen was used. It contained a hole in front of each eye, offering a view on two vertical and one horizontal target, drawn on an opaque board 2 cm behind the projection screen. For each eye, subjects aligned three projected lines with the drawn lines on the board. From this, the position of the rotation center of each eye could be derived, which was used for the correct projection of the flow scene (for a detailed description of the calibration procedure, see Supplementary Material). **(C)** Each panel shows the projection of the 3D cloud of dots, received by the observer as a flow-field here projected on a hemisphere concentric with the head. For clarity, in this figure the projected points were located on a regular grid of visual directions, but in the actual stimuli the points were in random visual directions about the head. The ensemble of motion vectors of individual elements of the flow scene depict T (left) and RT (right) motion. A perturbation of the projected motion vector's direction in 3D was introduced in the noise conditions (*N*_30_, *N*_60_). The vector was rotated about an axis (T motion, dotted line) perpendicular to the tangent plane to the sphere as illustrated for the unperturbed vector (arrow with shadow) into the perurbed vector (arrow without shadow). The same perturbation was applied to RT flow but is not drawn here. This implies that component of motion toward the head was unaffected. Direction and magnitude of the rotation were randomly chosen for each element of the scene.

As explained below in detail, we used a 2 × 3 × 2 factorial blocked design in which we combined two types of self-motion (T: translational motion and RT: rotational + translational motion) with three categorical motion types inflicted by the degree of noise (self-motion, distorted self-motion and object-motion). We presented the flow patterns monocularly (not used for analysis), synoptically (binocular viewing with zero disparity) and in stereo on a wide-field screen (~110 horizontal × ~100 vertical degrees field of view, with a maximum of ~80 × ~100° of binocular overlap), placed in close proximity to the head (Figures 1A,B). A detailed description of the calibration procedure and the wide-field projection set-up is provided in Supplementary Material.

#### View modes

Stimuli were presented to eight subjects in three different view modes: **monocular, synoptic**, and in **stereo**. During the monocular conditions, optic flow was presented solely to the left eye. The gray background ensured that luminance was kept constant between the eyes. However, we decided to exclude monocular presentation from analysis, for reasons mentioned below (see Monocular Presentation). In four subjects that were investigated in a second round, we excluded the monocular conditions from presentation.

During synoptic conditions, flow was presented to both eyes. The flow simulated self-motion as viewed from the ego-center (i.e., midway between the two eyes). This flow scene was presented identically to left eye and right eye. Hence, zero disparity was presented for all dot pairs.

For the stereo condition, the flow scene simulated motion with the same 3D scene properties, but projected onto either the left or right eye, and therefore contained full disparity information according to the points' simulated positions.

#### Simulated self-motion types

Two simulated self-motion conditions were presented.

1. The self-translation (**T motion**) condition: we simulated a forward motion on a straight path through the cloud of dots (speed: 1.0 m/s). The direction of gaze was aligned with the heading direction throughout the presentation.
2. The self-rotation + translation (**RT motion**) condition: we simulated a forward motion of 0.4 m/s in combination with an sinusoidal rotation. This simulation corresponds with self-motion along a strongly undulating path with the direction of gaze always aligned with the forward heading direction, which was constantly changing. Peak rotation velocity was 10°/s and peak amplitude 9.6°. The frequency was 1/6 Hz. The speed of forward motion was reduced by a factor 2.5 to compensate for a significant amount of motion energy added by the superimposed self-rotation. Both factors contribute to change the flow field from a translation-dominated to a rotation-dominated flow. Because the rotational flow does not convey depth information, while the translational component does, the T and RT flow conditions test the effect of rich and poor depth information from the flow field, respectively.

#### Noise/motion categories

The aim of the noise implementation was to create three categorical flow scenes: a **self-motion** pattern (**sm**, *N*_0_), a **distorted self-motion** pattern (**dsm**, *N*_30_), and a pattern of multiple independently moving objects (**mimo**, *N*_60_). The categorical distinction was validated by a perceptual study. Noise was implemented for both T and RT self-motion conditions by rotating the motion vector of each dot in the scene about the direction from the eye toward that dot (illustrated for T motion in Figure 1C). The angle of rotation of the motion vector was drawn from a uniform distribution with a width of 0, 30, or 60° (*N*_0_, *N*_30_, *N*_60_). The direction of rotation was clockwise or counterclockwise and randomly attributed to each point, doubling the effective width of direction randomization. This angle of deviation was fixed for each dot until it was refreshed. This manipulation degrades the pattern of self-motion without changing the magnitude/speed of the projected local motion vectors. Thus, the local motion energy was maintained across noise level.

Combining the different view modes, self-motion types, and motion categories, resulted in a total of 18 conditions. Movies of one monocular and all binocular conditions can be viewed in Supplementary movies 1–13.

### Scan parameters

The MR data acquisition was conducted on the 3 Tesla TIM Trio Siemens scanner at the Donders Centre for Cognitive Neuroimaging (Nijmegen, The Netherlands). For each subject, we obtained a high-resolution full-brain anatomical scan with a 32-channel head coil (T1-weighted MPRAGE, 192 slices, 256 × 256 matrix, resolution of 1 × 1 × 1 mm). For the experimental scans, the bottom half of the head coil was used, with only 20 channels to enable the wide-field screen presentation. Functional scans were obtained with an in-plane resolution of 2 mm iso-voxel and a slice distance of 2 mm (0.2 mm gap thickness; T2^*^-weighted; single-shot echoplanar imaging; 32 slices; repetition time (TR), 2 s; echo time, 30 ms).

### Functional localizers

In a separate session, we performed both retinotopic mapping and MT^+^ localization in all subjects. In 4 subjects, the localizers were already performed for a previous experiment. The stimuli were presented monocularly to these subjects. To ensure correspondence, the localizer stimuli for the remaining 8 subjects were also presented monocularly (left eye only). To demarcate visual areas V1–V7, we used standard retinotopic mapping techniques (Sereno et al., 1995). For the polar angle mapping, the subject was asked to fixate at the central fixation point while a black/white checkerboard “wedge” stimulus (wedge width, 60°) rotated counterclockwise about the center over the visual field at one revolution in 64 s. For the eccentricity mapping, subjects maintained straight ahead fixation while a radial black/white checkerboard ring moved from inner to outer visual field (maximum eccentricity, 60°) at a speed of 2°/s. Both ring and wedge consisted of a black/white alternating checkerboard (2 Hz) and were scaled by eccentricity in accordance with the cortical magnification factor (Rovamo and Virsu, 1979).

For the MT^+^ localizer, a blocked design was used. Full-field optic flow, flow in the left hemifield, flow in the right hemifield, and a rest condition (full-field static random-dot pattern) were alternated. The total duration of the run was about 6 min. Each block lasted for 18 s, and all three conditions were presented 3 times during the run. For the left hemifield and right hemifield conditions, flow was presented beyond 15° eccentricity; the remaining visual field was filled with a static random dot pattern. In all flow conditions, the flow simulated a forward motion of 2 m/s.

The retinotopy data were analyzed by a cross-correlation analysis between the BOLD activation and the stimulus. Direction reversals of the phase lag of the BOLD signal were taken as the borders of the main visual areas. These borders were drawn by eye on a phase-colored flat map representation. V1, V2, V3, V3ab, V6, and V7 were identified and demarcated based on their retinotopic organization. Area V6 was defined as a region medial to V3ab and V7 that contained a representation of the entire contra-lateral visual field and an eccentricity map (Pitzalis et al., 2006). In all subjects, V6 was located on the posterior branch of the Parieto-Occipital Sulcus (POS).

MT^+^ was defined as all voxels that responded significantly to full field motion stimulation within the dorsal part of the inferior temporal sulcus. We presented ipsi-lateral and contra-lateral flow to distinguish putative human MT and putative human MST in our subjects, as previously reported (Dukelow et al., 2001; Huk et al., 2002). We note that the parcellation of MT^+^ is more complex than this division; human MT and MST likely comprise multiple functional regions (Kolster et al., 2010) with possibly different contributions to heading and flow parsing, but the extended retinotopy mapping needed for this classification go beyond the scope of this study. However, the older distinction between two parts of MT^+^ has a functional meaning in that it separates the MT^+^ part with contralateral receptive fields from a sub region with bilateral receptive fields. Likely, these groups of cells have different functional contributions to optic flow analyses described here. Hence, we considered the localizer meaningful, and named the contra-lateral responsive and bilateral responsive part as MT^+^/contra (MT^+^/c) and MT^+^/bilateral (MT^+^/b) respectively.

MT^+^/c and MT^+^/b were defined as sub-regions of MT^+^. Area MT^+^/b was defined as the anterior part of MT^+^, including all contiguous voxels that responded to ipsi-lateral flow presentation (Dukelow et al., 2001; Huk et al., 2002). Area MT^+^/c was defined as a region containing contiguous voxels that responded to contralateral, but not ipsi-lateral, flow (at least *p* < 0.05, uncorrected).

We identified areas putative 2v (p2v), the ventral cingulate sulcus (CSv), and the ventral intraparietal area (VIP) based on their response to self-motion. We used a contrast between wide-field moving dots and static dots (Arnoldussen et al., 2011) to identify these areas and compared the resulting ROIs with previously reported findings that used the contrast between self-motion compatible and self-motion incompatible flow (Cardin and Smith, 2010). The areas were identified bilaterally based on the averaged response to T and RT motion combined. The *p*-value of the resulting statistical map was lowered until separate blobs of contiguous voxels of about 200 mm^2^ were clearly visible on an inflated map representation of each subject's left and right hemisphere (at least *p* < 0.01, uncorrected). In this way, the areas were identified in all hemispheres (16/16). As the Talairach coordinates closely resemble those of Cardin and Smith (2010) (Table 1), we used their nomenclature for these areas noting their provisory status (Cardin and Smith, 2011).

**Table 1:** **Mean Talairach coordinates of the ROIs in this study (± *SD* across subjects, *n* = 12)**.

ROI	Left			Right		
	***x***	***y***	***z***	***x***	***y***	***z***
V3ab	−17 ± 4	−87 ± 4	21 ± 6	18 ± 5	−85 ± 4	20 ± 6
V6	−14 ± 4	−77 ± 3	28 ± 6	14 ± 2	−75 ± 3	28 ± 5
V7	−24 ± 3	−78 ± 5	25 ± 6ß	25 ± 3	−79 ± 4	26 ± 5
MT^+^/c	−40 ± 3	−74 ± 5	10 ± 6	41 ± 3	−70 ± 5	5 ± 6
MT^+^/b	−43 ± 3	−67 ± 5	6 ± 5	44 ± 3	−63 ± 4	3 ± 3
CSv	−11 ± 1	−27 ± 5	42 ± 3	11 ± 2	−27 ± 7	41 ± 3
p2v	−30 ± 3	−42 ± 4	52 ± 4	30 ± 3	−42 ± 7	50 ± 3
pVIP	−22 ± 4	−60 ± 7	51 ± 5	24 ± 5	−59 ± 5	50 ± 5

### Experimental procedures fMRI

The T and RT flow patterns were presented in two separate sessions. In total, 10–13 functional runs were collected in each subject. Each run lasted for about 5 min (4 min for the second subject group). Conditions were presented in a blocked design with 18 s (9 TR) per condition. For both T and RT flow, each run consisted of nine (subject 1–8) or 6 (subject 9–12) conditions (3 levels of view mode × 3 levels of noise, or 2 levels of view mode × 3 levels of noise). All conditions were interleaved by a synoptic static random dot pattern, for baseline. Each run started and ended with the synoptic pattern.

Half of the runs were presented in backward order to account for possible order effects. This pairing was done for runs of all three axes of simulated rotation. The starting condition of each run was preceded by 4 dummy TRs, which were not analyzed.

In the MRI sessions, we used three cardinal axes of simulated rotation. The axes were perpendicular to the semicircular canal planes. For each run, only rotation about one cardinal axis was presented.

### Fixation and task

Irrespective of the view mode, the fixation point was always presented binocularly at a simulated distance of 2 m in front of the head within the 3D scene. The visual projection setup did not allow for eye-tracking in the scanner, due to the close proximity of the screen to the head (±3 cm). To promote stable fixation during all conditions, the fixation point was continuously visible at a fixed position on the screen. Only the scene of dots changed during the transition to a new condition. Subjects were explicitly instructed to fixate as accurately as possible. To further promote fixation during the run, subjects performed a detection task on the fixation point, reporting whether during the presentation its size changed, by pressing a button. In about 40 percent of the trials (both flow and static trials, i.e., ~2 times per minute), the fixation point changed slightly in size for about 1 s, with a variable start time.

### Data analysis

Brainvoyager QX (version 2.6) was used for the analysis of all anatomical and functional images (Brain Innovation, Maastricht, The Netherlands). Anatomical images were spatially normalized according to the atlas of Talairach and Tournoux to obtain standardized coordinates for the ROIs.

Functional images were corrected for 3D head motion and slice acquisition timing. Subsequently we applied to the resulting time courses linear trend removal and high-pass filtering by fitting a General Linear Model (GLM) with Fourier lower frequency cut-off set at two cycles per run. No spatial smoothing was applied to the functional data.

We examined the BOLD responses to the experimental conditions by application of a GLM for each subject separately, with a separate regressor for each individual condition. T and RT motion runs were analyzed separately. For the RT motion conditions, data were pooled across simulated axes of rotation. For each of the independently defined ROIs, the contribution of each regressor (quantified by beta-values) was obtained by a multi-study GLM on all condition repetitions. The resulting beta values (18 conditions, 11 ROIs) were used for subsequent multi-subject statistical analyses (i.e., random effects analyses).

A Repeated Measures Analysis of Variance (RM ANOVA) was used to evaluate interaction and main effects between stimulus factors. For any discussed effect, more complex interactions between factors were evaluated, and reported if statistically significant (*p* < 0.05). More specific analyses were performed using paired *t*-tests or, broken down RM ANOVA tests at different levels of one stimulus dimension. For such follow-up tests, Bonferroni correction was applied, correcting for the increased Family-wise error rate at multiple comparisons within ROIs. For example, within a ROI the criterion *p*-value for significance of effects for T and RT motion tested separately is 0.05/2 = 0.025. This level of significance is reported as *p* < 0.05 (BONF). We also report estimates of effect size for some analyses, primarily for comparison across ROIs. It was defined by partial eta squared, defined as:

$$p\eta^{2}=\frac{SS_{\text{effect}}}{SS_{\text{effect}}+SS_{\text{error}}}$$

*SS*_effect_ and *SS*_error_ refer to the Sum of Squares of effect and error, respectively.

### Monocular presentation

BOLD responses to all motion categories and self-motion patterns were also measured during a monocular presentation of the flow. We aimed at a comparison of monocular and synoptic presentation of flow to learn about binocular summation of motion signals in visual cortex. However, the difference between synoptically and monocularly presented flow fields might be overestimated relative to the difference between synoptic and stereo presentation, because the BOLD response to the synoptic static condition might be higher than a monocular static condition (which we did not present). Hence, we cannot fully attribute the difference between synoptic and monocular presentation to binocular summation of motion signals and we therefore excluded the monocular view mode condition from all analyses.

For the final 4 subjects, we excluded the monocular conditions from the experimental design.

### Perceptual study

We validated our categorical distinction between self-motion and object-motion stimuli in a perceptual study, in which five subjects rated their percepts on a self-motion and object-motion scale. In a 45-min session, separate from the fMRI data-acquisition session, subjects were asked to make perceptual judgments about the different conditions presented in the MRI session (monocular conditions were excluded). The study was performed within a dummy scanner at the Donders Centre for Cognitive Neuro-imaging (Nijmegen, The Netherlands). Projection set-up and stimulus properties were identical to those in the MRI session (Figures 1A,B), except for the order and duration of the conditions, and the addition of dot lifetime as a stimulus condition. The asynchronous lifetimes of individual points within the scene were either 400 or 2000 ms. Static conditions were not presented; a manually released pause was introduced between the different conditions to allow time for the subject to make judgments. Also, conditions were presented only for 1 cycle (=6 s). All subjects were able to make confident judgments based on this stimulus exposure time. All subjects were given the same written explanation of the task. If needed, the explanation was read aloud again during the experiment. Subjects were asked to make 2 judgments, giving a measure from 1 to 5 about:

1. The saliency of the self-motion percept. “How would you rate the percept in terms of self-motion in a range from 1 to 5, 1 being there is much movement, but each object follows an independent trajectory and 5 being there is much movement, but the movement is coherently moving relative to me, or I am moving relative to the objects in the world?”
2. The saliency of independent objects moving. “How would you rate the percept in terms of objects or random motion in a range from 1 to 5, 1 being there is much movement, but individual moving objects can hardly be discerned from the moving mass, and 5 being there is much movement, but individual moving objects can easily be discerned from the moving mass?”

## Results

### Perceptual study results—noise conditions evoke distinct motion percepts

**Figure 2:**
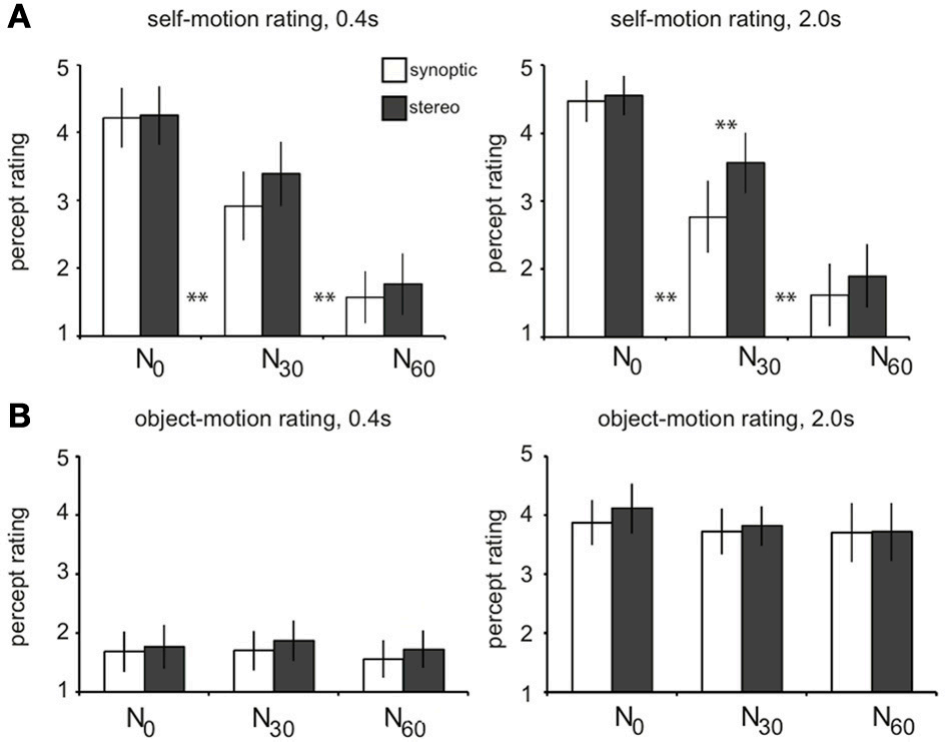
**Results of the perceptual study**. Subjects (*n* = 5) viewed the wide-field flow patterns in a dummy scanner setup while fixating a stationary central ring and judged each stimulus condition on a self-motion and object-motion scale (from 1 to 5). Results are averaged over subjects. **(A)** On the self-motion scale, subjects judged the *N*_0_ conditions as self-motion (mean: 4.4), the *N*_30_ conditions as degraded self-motion (mean: 3.2), and the *N*_60_ conditions as no self-motion (mean: 1.7). Adjacent levels of noise differed significantly, for both short and long lifetimes [two-sided paired *t*-tests: *N*_0_ > *N*_30_: lifetime_400_/lifetime_2000_, *t*_(4)_ = 5.0; 7.4, ^**^p < 0.01; *N*_30_ > *N*_60_: lifetime_400_/lifetime_2000_, *t*_(4)_ = 9.1; 19.4, ^**^p < 0.01]. There was no overall difference in self-motion rating between RT and T motion (*p* = 0.3). For longer lifetimes, degraded self-motion was rated on the self-motion scale significantly higher when presented in stereo compared to synoptic presentation (lifetime_2000_: stereo_30_ > synoptic_30_: ^**^p < 0.01; lifetime_400_: stereo_30_ > syn_30_: *p* = 0.17). **(B)** On the object-motion scale, subjects judged conditions with a shorter dot lifetime (400 ms) as noise, being judged significantly lower than the conditions with longer lifetime (2000 ms) that were judged as pursuable objects that move through the 3D scene (^**^p < 0.01). There was no significant difference between the adjacent noise conditions (all: *p* > 0.05). Error bars represent between-subject standard error of the mean (SEM).

For increasing noise level, subjects judged the salience of the self-motion percept from strong (*N*_0_) down to virtually absent (*N*_60_, Figure 2A). Adjacent levels differed significantly, and there was no overall difference of rating between RT and T selfmotion [not shown; two-sided paired *t*-test, *t*_(4)_ = 1.2, *p* = 0.31]. Remarkably, subjects gave a higher self-motion rating for degraded self-motion (*N*_30_), if presented in stereo. This was true, only for the longer lifetime presentation [one-sided paired *t*-tests: stereo_30_ > synoptic_30_for lifetime_2000_: *t*_(4)_ = 4.80, *p* < 0.01; for lifetime_400_: *t*_(4)_ = 2.25, *p* > 0.05]. Also, only points with long lifetime (2000 ms), were judged as “trackable” by eye pursuit or by attention, irrespective of noise level (average rating: 3.8; Figure 2B). For much shorter lifetime (400 ms), motion was judged c ccc haotic and offered no basis for object directed attention (average rating: 1.7).

Thus, for the long lifetime, our noise manipulation turned the percept in different motion categories: from pure self-motion (**sm**, *N*_0_) into degraded self-motion (**dsm**, *N*_30_), to a percept of just multiple independently moving objects (**mimo**, *N*_60_). Below we will use these perceptual categories rather than the noise levels to investigate how the contrasts in the BOLD responses relate to these distinctly different percepts.

### fMRI results

#### ROI identification

**Figure 3:**
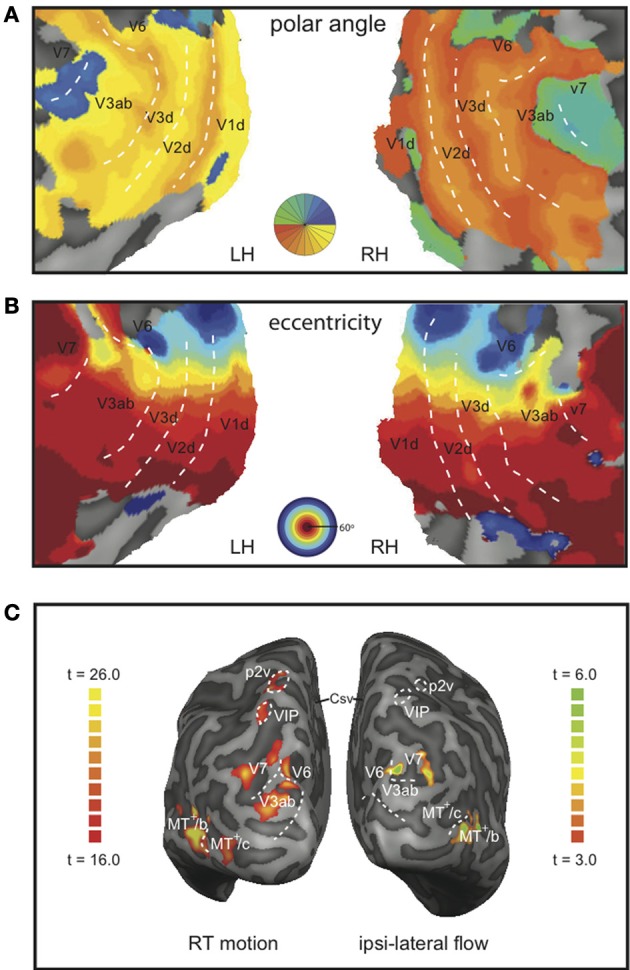
**Representative example of the demarcation of the ROIs and the BOLD responses**. Flat map representation of the dorsal-occipital part of the left and right hemisphere of a single subject with a color map of the phase angle mapping **(A)**, or eccentricity mapping **(B)**; both: correlation coefficient *r* > 0.3. Dotted lines demarcate the main visual areas. **(C)** Inflated representation of the left and right hemisphere of the same subject. On the left hemisphere, the result of a GLM contrast between all motion conditions vs. static is depicted (RT motion runs). The right hemisphere shows the result for a contrast between right-hemifield flow vs. static (MT^+^-localizer runs).

Lower tier visual areas (V1, V2, V3) and 8 additional motion-sensitive areas (V3ab, V6, V7, MT^+^/c, MT^+^/b, CSv, p2v, VIP) were identified in all 7 subjects bilaterally, using retinotopy (Figures 3A,B) and standard localizer procedures (see Materials and Methods section for details, Figure 3C). Talairach coordinates and anatomical locations (Table 1) were very similar to those reported previously (Cardin and Smith, 2010). Only in a minority of the hemispheres, we were able to further subdivide the full contralateral hemifield representation anterior to V3 that defines v3ab into V3a and v3b as described before (Larsson and Heeger, 2006; Wandell et al., 2007). Therefore, we defined the ROI as V3ab, encompassing both regions. Area p2v was located in all subjects within the dorsal portion of the postcentral sulcus. VIP was always located within the fundus of the intraparietal sulcus.

#### Global motion energy equated responses in lower tier visual cortex

**Figure 4:**
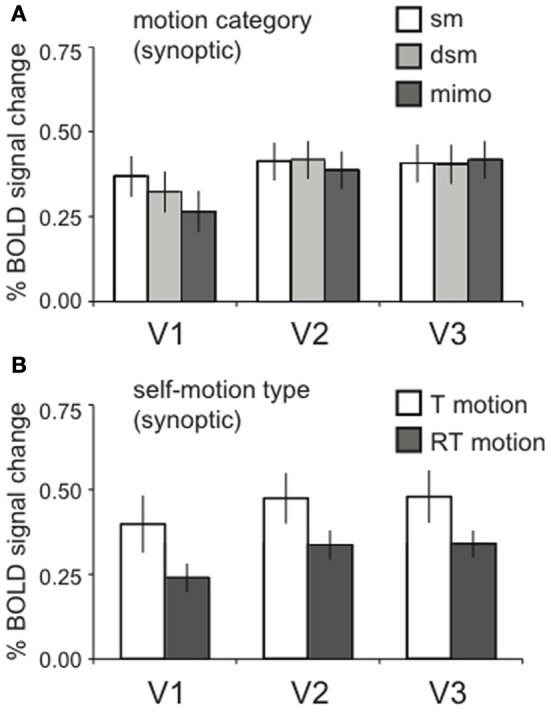
**Lower visual areas response to motion energy. (A)** The averaged BOLD response across motion category dimension, for synoptic presentation. Within V1, V2, and V3, there was no response difference between motion categories [RM ANOVA, V1, V2, V3 all *F*_(2, 22)_ < 2.07; *p* > 0.05]. **(B)** The averaged BOLD response for the two self-motion types (RT motion, T motion), for synoptic presentation. There was no effect of self-motion type RM ANOVA, V1, V2, V3: all [*F*_(1, 11)_ < 4.42, *p* > 0.05], though a trend for a larger response to T motion can be observed. Error bars represent SEM.

The local motion energy was identical across the motion category dimension. Thus, we expected equal BOLD activation in motion areas that process motion locally for synoptic stimuli (Van Essen and Gallant, 1994; Grill-Spector and Malach, 2004). Indeed, no significant effects of motion category were found in lower-tier visual areas V1, V2, and V3 [RM ANOVA, V1, V2, V3 all *F*_(2, 22)_ < 2.07; *p* > 0.05, Figure 4A].

We presented optic flow with translational motion (**T motion**) and with translational motion plus a large rotational component (**RT motion**). The comparison between these different motion types is more difficult to interpret because RT and T motion conditions were collected in different sessions. Moreover, the addition of a large rotational component increases the local motion energy. To alleviate the latter complication, we reduced the speed of the simulated forward translation, This compensates globally for the addition of the rotational flow, but cannot restore equal local motion energy across the visual field. However, because we report BOLD signals across ROIs of both hemispheres, the integrated response reveals the response to the entire visual field, and our global compensation may succeed. We remark that there was a trend for higher responses to T than to RT motion in the lower tier areas V1–V3 (Figure 4B) but, this difference was not statistically significant [RM ANOVA, V1, V2, V3: all *F*_(1, 11)_ < 4.42, *p* > 0.05].

Given these results, main and interaction effects between view mode and motion category in higher-tier motion areas can be attributed to a functional specificity of the ROI rather than local motion processing constraints by the lower tier areas that are passed on to higher tier areas. This is also true for effects of self-motion type, although the trend we mention above precludes strong conclusions when higher tier motion regions have a preference for T motion. In the subsequent results, we report specific effects within the ROIs. Their full response patterns over all stimulus conditions are presented in Supplementary Figure 1.

#### Self-motion selectivity?

In previous fMRI studies, V6, VIP, CSv, and p2v showed a stronger response to self-motion compatible optic flow (1 large patch of flow) than to self-motion incompatible flow (9 small patches of flow; Wall and Smith, 2008; Cardin and Smith, 2010). We perturbed the self-motion pattern by random rotation of each motion vector about its visual direction. This preserved local motion energy for the synoptic condition while the self-motion information was degraded, because the flow pattern was disrupted. Is this reflected in the BOLD signal?

We decided to analyse the effect of noise on the BOLD response to self-motion only for the stimuli for which subjects did report a self-motion percept in our perceptual study, i.e., motion categories **sm** and **dsm**. We analyzed the BOLD responses separately for T and RT self-motion types, because the direction of self-motion perception is much more robust to noise for pure self translation (T) than for Rotation+Translation flow (RT) (Lappe et al., 1999).

We tested all ROIs for the effect of degrading self motion information. V6 and CSv showed a significant drop in BOLD response from self-motion (sm) to degraded self-motion (dsm) stimuli for the synoptic, RT flow conditions [one-sided paired *t*-test: *t*_(11)_ = 2.78; 3.69, *p* < 0.05, BONF]. No region showed such decrease for synoptic, T flow [all ROIs: *t*_(11)_ < 0.65, *p* > 0.05, BONF, Table 2: general sm preference].

**Table 2:** **Overview of the primary effects tested and the *F*, or *t* statistics for the different ROIs**.

	General sm preference **synoptic sm, synoptic dsm**	Binocular contribution to self-motion (syn, stereo)^*^ (sm, dsm)	General mimo preference **sm, mimo**	Binocular contribution to object-motion (syn, stereo)^*^ (sm, mimo)	**mimo stereo > mimo synoptic**
	RT motion	RT motion	RT + T motion	RT + T motion	RT + T motion
ROI	*t*_(11)_ =	*F*_(1, 11)_ =	*F*_(1, 11)_ =	*F*_(1, 11)_ =	*t*_(11)_ =
v3ab	0.83, *p*^B^ = 0.21	2.14, *p*^B^ = 0.17	6.39, ^*^p = 0.03	4.27, *p* = 0.63	
v6	2.78, ^*^p^B^ = 0.01	14.64, ^**^p^B^ = 0.003	1.06, *p* = 0.33	9.35, ^*^p = 0.01	3.77, ^**^p = 0.003
v7	1.00, *p*^B^ = 0.17	0.73, *p*^B^ = 0.41	3.38, *p* = 0.09	1.93, *p* = 0.19	
mt^+^/c	−0.75, *p*^B^ = 0.76	0.30, *p*^B^ = 0.60	19.42, ^**^p = 0.001	1.51, *p* = 0.24	
mt^+^/b	0.67, *p*^B^ = 0.26	5.08, *p*^B^ = 0.05	24.67, ^***^p < 0.001	2.21, *p* = 0.17	
CSv	3.69, ^**^p^B^ = 0.002	8.85, ^*^p = 0.01	3.55, *p* = 0.09	5.94, ^*^p = 0.03	−0.34, *p* = 0.63
p2v	2.12, *p*^B^ = 0.03	4.75, *p*^B^ = 0.05	2.48, *p* = 0.14	3.01, *p* = 0.11	
VIP	2.02, *p*^B^ = 0.03	3.58, *p*^B^ = 0.09	0.11, *p* = 0.75	3.29, *p* = 0.10	

Top row gives a description of the effect tested below for each ROI, and the viewmode and motion category conditions included (in bold); second row shows which motion type conditions are included. Final column shows no description, but only the effect tested (mimo stereo > mimo synoptic). Effects were considered significant if p < 0.05 (shown as:

* p < 0.05,

** p < 0.01, or

*** p < 0.01).

If needed, the significance threshold of p < 0.05 was corrected for multiple testing by dividing it by the number of repeated tests performed within a ROI (i.e., Bonferroni corrected p value, p^B^, see also Materials and Methods).

Hence, offering just synoptic information, moderate noise (dsm) reduced the BOLD signal. Note that noise had this effect only, if the depth cue in the flow was reduced already due to the addition of rotation (RT). Noise addition did not affect the BOLD signal when there was a strong depth cue from the pure translational flow (T).

#### Binocular contribution to self-motion processing

In our perceptual study self-motion ratings declined with increasing noise level, both for stereo and synoptic presentation. For degraded self-motion a significant difference occurred between the different view modes. Then, the self-motion rating was significantly higher for the stereo condition (Figure 2A). This is reminiscent of previous perceptual studies using RT stimuli. In those studies stereo presentation improved heading discrimination only if noise was added to the flow (Van Den Berg and Brenner, 1994b; Macuga et al., 2006).

Together these perceptual results indicate that visual self-motion signals and disparity signals fuse to obtain a more robust detection of self-motion. Can we find BOLD responses in any of our ROIs, pointing to such fusion of depth from motion parallax in the flow and binocular disparity?

Specifically, heading detection becomes non-trivial when gaze rotation disrupts the coincidence between focus of expansion and heading direction. For that reason, and because the overall forward speed was lower for RT than T conditions, we expected that addition of noise to RT motion hampers areas involved in heading detection especially. Then, BOLD responses may be less affected by noise for stereo than for synoptic RT motion.

For T self-motion, effects of viewmode may be marginal, because random motion does not bias heading.

**Figure 5:**
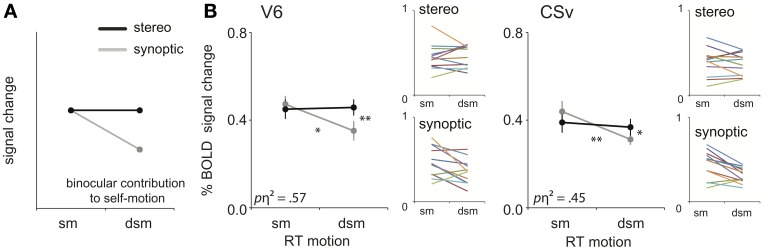
**Binocular processing of self-motion. (A)** Prediction of the response pattern for a ROI that is involved in binocular processing of self-motion. When the retinal flow simulates complex self-motion (forward motion in combination with large rotational motion), degradation of that pattern's structure by noise reduces the self-motion percept (synoptic condition), unless stereo information helps to uncover the depth order of the scene (Van Den Berg and Brenner, 1994a). **(B)** The subject-averaged responses of V6 and CSv for sm and dsm, for the two viewmodes (synoptic, stereo). Small color graphs present individual results. Symbols represent *t*-test outcomes as described in text: ^*^p < 0.05, ^**^p < 0.01. *p*η^2^ = partial eta squared. Error bars represent SEM.

We assessed these predictions, by testing our ROIs for **binocular contribution to self-motion processing** (Figure 5A), i.e., a significant interaction between view mode (synoptic, stereo) and the degradation of self-motion information (**sm**, **dsm**).

For RT flow, the interaction effect was significant in area V6 and CSv [RM ANOVA, V6:/CSv *F*_(1, 11)_ = 14.64; 8.85 *p* < 0.01, BONF; Figure 5B, Table 2: binocular contribution to self-motion]. A trend of the effect was observed in MT^+^/b, p2v, and VIP, but this did not reach significance [*F*_(1, 11)_ = 5.08; 4.75; 3.58, *p* = 0.09; 0.14; 0.17, BONF].

As stated earlier, V6 and CSv, but not MT^+^/b, showed a decrease in response from synoptic sm to dsm. In addition, within V6 and CSv, the BOLD response to stereoscopic presentation was larger than synoptic presentation for degraded self-motion [one-sided paired *t*-test, *t*_(11)_ = 4.04; 2.95, *p* < 0.05, BONF]. There was no such difference for self-motion [one-sided paired *t*-test: *t*_(11)_ = 0.65; 1.44, *p* > 0.05, BONF]. Therefore, the interaction effect observed in V6 and CSv truly points to binocular contribution to self-motion processing.

For T flow, a similar analysis yielded no such significant interaction effect for any ROI [RM ANOVA, all ROIs: *F*_(1, 11)_ < 0.40, *p* > 0.05, BONF]. Hence, stereo signals enhanced the BOLD response selectively for the degraded self-motion condition, but only for the complex flow with rotation added to the translation.

#### Preference for multiple independently moving objects

**Figure 6:**
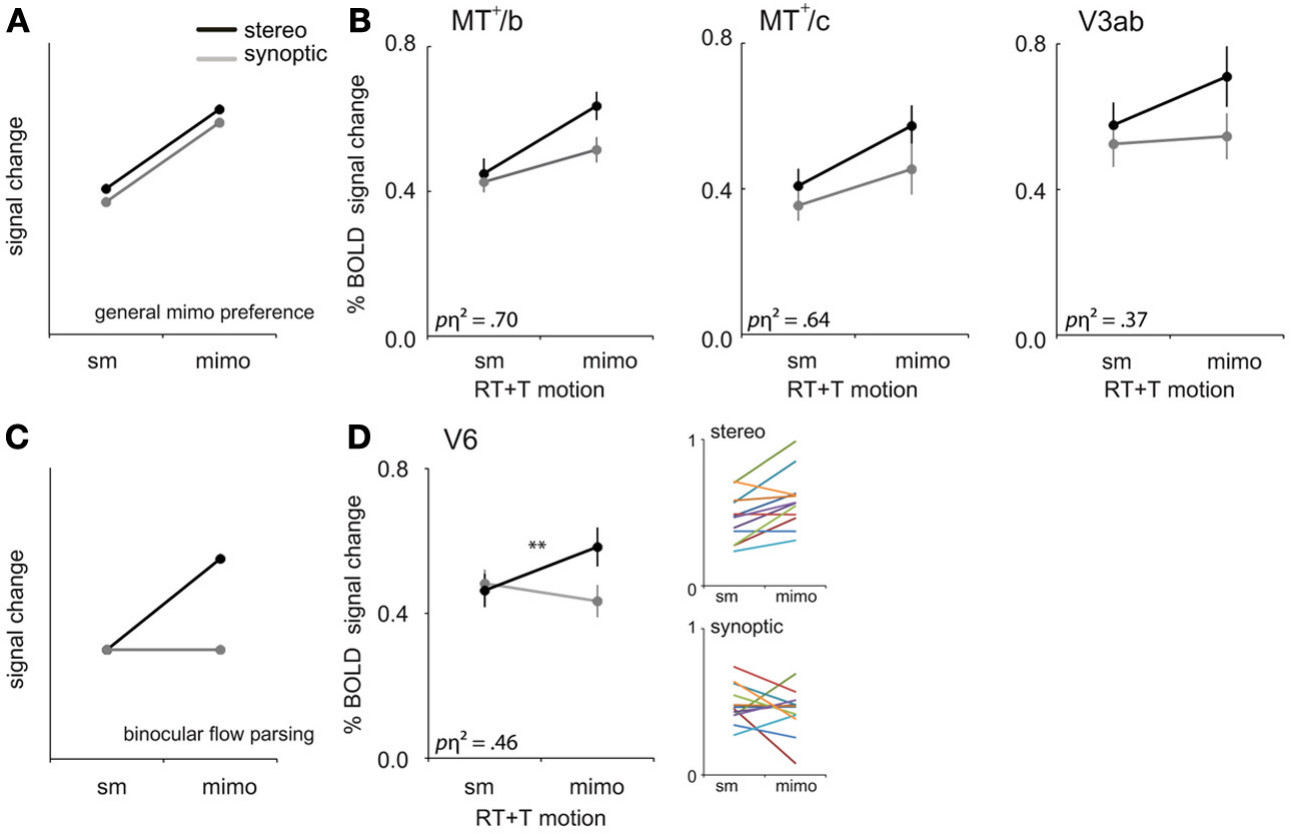
**mimo preference. (A)** Prediction of the response pattern for a ROI that shows a general preference for multiple independently moving objects (mimo) compared to self-motion (sm) that is not affected by viewmode. **(B)** Responses of V3ab, MT^+^/c, and MT^+^/b to sm and mimo for synoptic and stereo presentation, for RT and T motion combined. **(C)** Prediction of the response pattern for a ROI that is involved in binocular processing of multiple moving objects. Such a region will respond most strongly if more objects move independently in stereo. Stereo signals may help to extract 3D object motion trajectories that are ambiguous in retinal coordinates. **(D)** Responses of V6 to **sm** and **mimo**, for RT and T motion combined. Small color graphs present individual results. Symbols represent *t*-test outcomes as described in text: ^**^p < 0.01, *p*η^2^ = partial eta squared. Error bars represent SEM.

In all our synoptic conditions the local motion energy and the number of objects remained the same. Yet, the percept changed from coherent optic flow depicting self motion (**sm**) to all points moving independently (**mimo**) without a self motion percept, at the highest noise level. For which ROIs did the BOLD response reflect this dramatic change of the amount of independently moving objects (i.e., a general mimo preference, Figure 6A)?

We observed a larger BOLD response to **mimo** compared to **sm** in V3ab, MT^+^/c, and MT^+^/b [RM ANOVA, *F*_(1, 11)_ = 6.39; 19.42; 24.67, *p* < 0.05, Figure 6B, Table 2: general mimo preference] that was *in*dependent of viewmode [interaction effect: *F*_(1, 11)_ < 4.51, *p* > 0.05].

For MT^+^/b only, the response difference for motion category did depend on motion type i.e., a significant interaction effect between motion category and motion type [*F*_(1, 11)_ = 4.97, *p* < 0.05]. Additional broken down RM ANOVA's revealed that the response in MT^+^/b increased from **sm** to **mimo**, for T motion [*F*_(1, 11)_ = 45.00, *p* < 0.001, BONF] but not for RT motion [*F*_(1, 11)_ = 1.48, *p* = 0.50, BONF].

Overall, these 3 ROIs showed a preference for multiple independently moving objects independent of viewmode (Figures 6A,B: general mimo preference), which was not present in the lower tier visual areas (V1-V3, Figure 4A).

#### Binocular preference for multiple independently moving objects

Human flow parsing performance is better for stimuli with binocular cues (Warren and Rushton, 2009a,b). Stereo signals also help to perceive the depth component of a moving object (Gray and Regan, 2000), and thereby potentially its relative motion in depth with respect to other objects in the scene. Thus, finding objects that are independently moving in the flow and extracting their motion direction in depth, profit from binocular information. However, these perceptual studies refer to single objects. Therefore, one cannot immediately infer that the same properties will hold necessarily for a multitude of objects as in our study. Yet, we wondered if stereoscopic depth cues would affect the BOLD response to multiple independent moving objects (**mimo**), even in areas that do not show a preference like area MT^+^/b. In other words, we looked for ROIs that show a **binocular contribution to flow parsing**, i.e., a contribution of stereo to separate a moving object from the multitude of other moving objects in the scene (Figure 6C).

First, we established that interactions between viewmode and self-motion type were not significantly different between self-motion types i.e., no significant viewmode × motion category × self motion type interaction [all *F*_(1, 11)_ < 0.36, *p* > 0.05]. Next, we pooled T and RT motion conditions and tested if any of the ROIs showed a significant interaction between view mode (stereo, synoptic) and motion category (**sm**, **mimo**). V6 and CSv showed such a significant interaction [RM ANOVA, *F*_(1, 11)_ = 9.35; 5.94, *p* < 0.05; other ROIs: *F*_(1, 11)_ < 4.27, *p* > 0.05]. However, only in V6, the response to stereo motion increased from **sm** to **mimo** [paired *t*-test, V6: *t*_(11)_ = 3.77, *p* < 0.01; CSv: *t*_(11)_ = −0.34, *p* = 0.63, Table 2: mimo stereo > sm stereo]. Thus, we found that only within V6, a pattern of BOLD responses that indicate a **mimo** preference that is dependent on stereo cues (Figure 6D).

#### Main effects of disparity and self-motion type

Finally, we investigated potential main effects of view mode and self-motion type (T, RT motion) on the BOLD signals.

**Figure 7:**
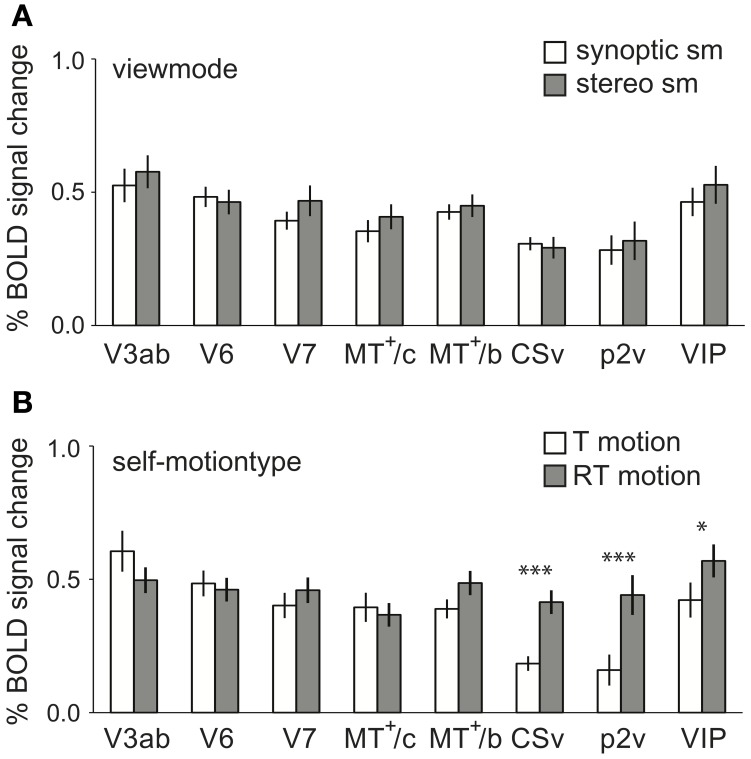
**Main effects of self-motion type and viewmode across subjects. (A)** The averaged BOLD response within the ROIs at different levels of viewmode (synoptic, stereo, only sm) and self-motion type (RT motion and T motion, sm data). No ROI showed a larger response to stereo presentation than to synoptic presentation of the stimuli. **(B)** The averaged BOLD response within the ROIs for sm at different levels of self-motion type (T motion, RT motion), pooled over viewmode (synoptic, stereo). CSv, p2v, and VIP showed a significantly larger response to motion with a strong rotational component (^*^p < 0.05, ^***^p < 0.001, *p*η^2^ = 0.69; 0.70; 0.39). Error bars represent SEM.

Regarding view mode, we found no significantly larger response to stereo self-motion than to synoptic self-motion in any of our ROIs [RM ANOVA, *F*_(1, 11)_ < 2.10, *p* > 0.05; Figure 7A].

Is there a general preference for one of the two self-motion types (RT and T motion, sm data only), irrespective of view mode (synoptic and stereo)? We found a strong preference for RT motion in CSv, p2v, and VIP [RM ANOVA, *F*_(1, 11)_ = 24.51; 25.01; 6.91, *p* < 0.05, see Figure 7B]. The observed preference is opposite from the trend observed in V1–V3.

## Discussion

Motion parallax and disparity signals (Rogers and Graham, 1979; Qian, 1997) can each be a sufficient cue to depth, but psychophysical studies point to fusion of depth cues for shape perception (Rogers and Graham, 1982; Rogers and Collett, 1989; Uomori and Nishida, 1994; Landy et al., 1995; Bradshaw and Rogers, 1996; Ichikawa and Saida, 1996; Domini et al., 2006), and perceived motion in depth (Beverley and Regan, 1975; Regan, 1993). Neurophysiological (Nadler et al., 2008), and neuroimaging (Ban et al., 2012) studies both indicate an interaction of these cues in some dorsal motion areas for improved depth processing.

To investigate such interactions with fMRI, we exposed subjects to a set of wide-field optic flow stimuli arranged in three different dimensions:

- Simulated self motion; we presented flow in which the extraction the direction of self motion was easy (pure translation T) or difficult (Rotation + Translation, RT).
- Motion category; we added noise to degrade the pattern of flow, which caused three qualitatively different percepts: self motion, noisy self motion and a set of independently moving points.
- Viewmode; we presented the stimuli with natural disparity (stereo presentation) and zero disparity (synoptic presentation).

These dimensions probe interactions that occur between motion signals and binocular depth signals for self-motion perception, for the distinction between self-motion and the motion of independently moving objects (flow parsing), or the estimation of the depth component of the independently moving objects.

We ensured that different types of noise stimuli contained identical local motion energy. Only then, interactions between motion category (dimension b) and view mode (dimension c) in the BOLD signal can point to fusion of motion and stereo cues to depth in the higher tier motion areas. Indeed, the BOLD signals in lower visual cortex were constant across the motion category dimension (Figure 4A), while differences showed up in higher-tier motion areas.

We found interactions between monocular and binocular cues to depth in higher-order motion areas. These areas were different from those reported before (Ban et al., 2012) likely because our stimuli were entirely different and used wide-field motion patterns.

### Binocular processing of self-motion and object-motion

In area CSv and V6, stereoscopic presentation countered the drop of the BOLD response when noise and simulated self-rotation degraded the monocular depth information from the flow (Figure 5B). Also, stronger self-motion ratings were given when a degraded flow pattern was presented stereoscopically (Figure 2A) compared to synoptically. Both observations comply with earlier psychophysical work on heading perception from pure stereo cues (Macuga et al., 2006), and observations that stereo information makes heading detection more noise-tolerant in the presence of rotation (Van Den Berg and Brenner, 1994b). Because heading detection becomes non-trivial when gaze rotation disrupts the coincidence between focus of expansion and heading direction (Warren and Hannon, 1988), these perceptual observations suggest that the stereo signal contributes to the dissociation of translational and rotational components of flow. A special manipulation of the stereo signal in (Van Den Berg and Brenner, 1994b) showed that the stereo cue conveys depth order, to identify the most distant points which move primarily due to the self rotation (Koenderink and Van Doorn, 1987). The BOLD responses in CSv and V6 suggest that stereo signals enable these areas to support separation of the noisy flow in self-rotation and self-translation. To our knowledge, this is the first fMRI evidence that stereo information may support self-motion perception.

Another new interaction was revealed by a comparison of the BOLD responses to a noise-free self-motion pattern and a swarm of incoherently moving dots with the same local motion energy. Area V6 showed identical responses to these different motion patterns when presented synoptically. The addition of stereo cues did not affect the BOLD response to the self-motion pattern but it did raise the BOLD response to the swarm (Figure 6D). Because in the self-motion pattern all the dots follow non-intersecting trajectories, their separation from each other is already given without the stereo signal. In contrast, the intersecting trajectories of the elements of the swarm do constitute ambiguity (did the dots cross or did they collide?) which can be resolved by the stereo signal. Thus, the BOLD interaction in our study of V6 appears to line up with a disparity dependent response to the separate objects' motions in 3D in the monkey (Galletti and Fattori, 2003).

### V6: involved in extracting complex 3D motion

Our main findings concern area V6, which has only recently been identified in humans (Pitzalis et al., 2006). Human V6 is positioned on the posterior part of POS, and contains large receptive fields that cover the entire contra-lateral hemi field without an overrepresentation of the fovea (Pitzalis et al., 2006). It is highly responsive to visual self-motion (Pitzalis et al., 2010) and is strongly modulated by rotational signals of the eyes (Arnoldussen et al., 2011; Fischer et al., 2012b).

Recent fMRI findings showed a response preference of V6 for flow patterns that signal self-motion compared to self-motion incompatible, or random motion (Cardin and Smith, 2010; Pitzalis et al., 2010; Cardin et al., 2012; Helfrich et al., 2013; Pitzalis et al., 2013). However, these studies did not investigate stereoscopic conditions. By presenting both synoptic and stereo flow patterns, we were able to identify the component in the BOLD response of V6 due to stereo signals, which point to a response preference for multiple independent moving objects (**mimo**, Figure 6C). Our results show that the response pattern in V6 does not exhibit a simple self-motion preference, but that its motion type preference is dependent on the presence of disparity signals (i.e., view mode dependent, Figure 6D).

A recent fMRI study found, solely for V6, a higher response to flow patterns that contained a naturalistic disparity scene layout (i.e., disparity increases with eccentricity) than a non-naturalistic layout [i.e., larger disparities at lower eccentricities, (Cardin and Smith, 2011)]. We take this result a bit further as we report flow-disparity interaction patterns that are characteristic for both binocular parsing of the flow into individual object motions and binocular processing of self-motion (Figures 5, 6). Overall, our findings add to the notion that V6 plays a pivotal role in parsing complex motion into self- and object motion components.

### General mimo preference

MT^+^/c, MT^+^/b, and V3ab show a response preference for **mimo** compared to **sm** (Figure 6B). Recent imaging studies found that MT^+^ responds to both self-motion compatible flow and random motion (Smith et al., 2006; Wall and Smith, 2008; Helfrich et al., 2013) but, in contrast to our findings, weaker activation of MT^+^ by random motion was reported in these studies. Similarly, most studies (Braddick et al., 2001; Moutoussis et al., 2005), but not all (Pitzalis et al., 2013), reported a preferred response to coherent compared to random motion in v3a.

The use of a shorter dot lifetime (<1000 ms previously, compared to 2000 ms in this study) may have caused the different outcome. Perhaps more importantly, we used a more limited randomization of the motion direction in the current study (*N*_60_ compared to *N*_180_ for previously used random noise). This means that some self-motion information was still present in our case because each local motion vector was drawn from a distribution that is biased in the self motion direction. Although our subjects did not perceive self-motion at the highest noise level (Figure 2A *N*_60_), our **mimo** stimulus is not the same as random noise. That assertion would ignore the important fact that the **mimo** stimulus still contains a component of the self-motion stimulus in each local flow vector, which is absent in pure noise (*N*_180_).

Because of our construction of the incoherent motion stimulus, it is appropriate to describe the response in MT^+^/b&c and V3ab as a BOLD signal increase that is proportional to the mean deviation angle from the unperturbed self motion stimulus. Because that same angle of deviation is the primary measure to parse the moving object from the flow in a flow parsing experiment (Rushton and Warren, 2005), this suggests that the BOLD response may reflect a processing step in MT^+^/b&c and/or V3ab responding to that deviation angle, i.e., the flow parsing measure. An older human PET study used a very similar method of generating incoherent motion (Beer et al., 2002) and wide field presentation. This study also reported a locus with larger PET signal strength for incoherent than for coherent horizontal flow in the V5/MT region.

We suggest therefore that MT^+^/b&c may contribute to flow parsing, to identify objects that move in the world while the observer is moving him/her self (Rushton and Warren, 2005; Warren and Rushton, 2009a). If so, MT^+^/b&c should be using both monocular and stereo cues (Warren and Rushton, 2009b), and cues from other modalities (Calabro et al., 2011; Macneilage et al., 2012) to simultaneously characterize the self-motion pattern and deviations from that pattern. This notion means that MT^+^/b&c BOLD level would reflect the combination of a response to a self-motion pattern and the total amount of deviation relative to that pattern. This then could explain why the BOLD signal rises when the deviation angle rises, up to a noise level where the signal for the self-motion pattern vanishes (definitely for *N*_180_, because the deviation vanishes in the absence of a global pattern), which then results in a reduction of the BOLD signal even below the response to self motion. Thus, this hypothesis is at least qualitatively consistent with both our results and those of the Smith group (Smith et al., 2006). Further experiments are necessary, however, to fully test this idea.

### Sensitivity to self-motion with(out) simulated eye/head rotation

Area CSv is known to prefer rotational motion (Wall and Smith, 2008), resulting from simulated eye or head rotation (Arnoldussen et al., 2011; Fischer et al., 2012a). Here, we replicated CSv's preference for wide-field 3D rotational flow (Figure 7B) and showed that CSv uses stereo information for the processing of self-motion signals (Figure 5B). Interestingly, CSv also receives vestibular information (Bottini et al., 1994; Lobel et al., 1998; Bremmer et al., 2002; Cardin and Smith, 2010), as well as extra-retinal signals on eye rotation (Bremmer et al., 1999; Arnoldussen et al., 2011). Together, CSv appears well-suited to integrate visual, vestibular and eye movement signals into a multi-modal representation of head rotation. It remains to be seen whether the rotation preference makes CSv a core area for perception of self-rotation.

### Cue combination

Recently, fusion of depth cues has been found in V3B/KO using multivariate approaches (Ban et al., 2012). We took another approach, and looked for univariate BOLD signal differences between stimulus conditions, maximizing these differences by using wide-field stimulus presentation and abundant stereo cues (i.e., large range of (changing) disparity). Our approach may have lacked the high sensitivity of multivariate approaches for the detection of fusion in other ROIs, for example V3B/KO. Hence, we believe that fusion of depth cues takes place but is not limited to V6 and CSv.

Does a univariate interaction effect truly point to fusion? Using fMRI one cannot distinguish between the possibility that the interaction patterns resulted from activity of different neuronal populations rather than fusion at the level of a single neuron. The interactions that we report between viewmode and motion category can only be explained by sub-populations that share information on motion category and view mode and are therefore highly interdependent. Thus, both observed interaction effects point to fusion at the level of voxel populations of stereo and motion parallax cues to depth.

For V6, we found evidence of two distinct interaction processes between stereo and motion. We consider it plausible that these disparity-motion interactions differentiate spatially within V6. Such a sub-ROI functional differentiation has been shown for interactions between retinal and extra-retinal signals on self-motion (Arnoldussen et al., 2011).

### Caveats

#### Vection and attentional load

Could vection percepts have affected our results? On debriefing, subjects reported little or no vection. As became clear from our perceptual study subjects could easily classify motion as self-motion or object-motion. Thus, we believe that percepts of vection are not causative to the processing of self-motion information within dorsal motion areas.

The detection task on the size changes of the fixation point ensured that subjects' attention was directed to the fixation point and a relatively constant level of attention was directed to the stimulus irrespective of its visual content.

#### Vergence eye position

We were unable to measure eye movements using our visual projection system. To ensure minimal influence of eye movements on the results, we ensured that the fixation point in depth remained at a fixed binocular position across different stimulus conditions within a functional run. Also, we ensured, prior to scanning that subjects were able to easily fixate and fuse the fixation point. Finally, we found minimal BOLD response differences in lower visual areas for the different conditions arguing against condition-specific eye fixation errors. In sum, we think eye movements had minimal influence on the results reported here.

#### Cue conflicts

Some of our stimuli offered a visual-vestibular and/or intra-visual cue conflicts. Were these possibly affecting the interpretation of our results?

For natural self-motion, both translational motion and rotational motion are also signaled by the vestibular system. We presented simulated self-motion stimuli to observers with an immobilized head, possibly inducing a visual-vestibular conflict. The conflict is expected to be largest at the RT motion conditions, because the sinusoidal rotational self-motion is, for natural self-motion, continually accompanied with vestibular signals, in contrast to the quickly adapting signal for constant translational motion from the otoliths. Thus, the strong response to RT **sm** in CSv, p2v, and/or VIP might signal the cue conflict, rather than a preference for visual rotational flow *per se*. In the current study, we cannot distinguish between these two interpretations. In any case, the response properties of these ROIs point to an involvement in the analysis of rotational self-motion signals.

Secondly, psychophysical studies on depth cue combination of motion and disparity for veridical shape perception point to a perceptual dominance of the binocular cue during cue conflicts (Uomori and Nishida, 1994). Such a cue conflict might have occurred in our synoptic conditions, where the flow defines 3D motion within a volume, but the disparity cues defines positions motion on a Vieth-Muller torus at 2 m distance. The conflict is diminished strongly for vergence position approaching infinity (i.e., parallel viewing directions for the two eyes), because for geometric reasons disparity differences beyond about 6 m become vanishingly small meaning that disparity does not provide reliable information on distance, not that all points are located in a surface. We presented all flow elements at zero disparity to the eyes that were fixating a point at 2 m, introducing a rather small conflict. Indeed none of our subjects reported to see themselves approaching a flat plane in the synoptic conditions. Yet, strictly one cannot exclude that *main* effects of *viewmode* might be the result of the cue conflict rather than a genuine stereo increased response. Most importantly, however, these considerations do not apply for the interpretation of the *interaction* effects reported, because the reported cue conflict for the synoptic conditions were constant across the motion category dimension, whereas the percept and the fMRI activities clearly changed. In fact for the mimo preference comparison one could argue that the cue conflict is largest for the synoptic self-motion and the stereo mimo conditions and least for the stereo self-motion (both cues indicate a cloud with abundant depth) and the synoptic mimo (both cues indicate a cloud with degraded depth information). Thus, if cue conflict would determine the response one would expect response clusters for these two combinations rather than the response pattern we reported (Figure 6D).

## Conclusion

Motion parallax and disparity signals each provide important visual information about self-motion and object motion in depth. Here, we investigated how these depth cues interact in human motion areas for wide-field visual motion stimuli. We presented complex flow patterns with natural stereo information and with zero disparity, and found interactions between stereo and motion parallax that are dependent upon the type of motion. CSv and V6 were found to use stereo for heading perception during complex *self*-motion. V6 also appears to rely on stereo information for the processing of 3D *object*-motion when the flow provides poor depth signals from motion parallax. These findings advance the understanding of the involvement of these regions in the analyses of complex motion encountered in natural situations.

### Conflict of interest statement

The authors declare that the research was conducted in the absence of any commercial or financial relationships that could be construed as a potential conflict of interest.
