# Supplementary material for: Differential responses in dorsal visual cortex to motion and disparity depth cues
Source: Front Hum Neurosci. 2013 Dec 2;7:815. doi: 10.3389/fnhum.2013.00815 (PMC3857528; doi:10.3389/fnhum.2013.00815)
Supplement: Supplementary file 1 [file Data_Sheet_1.DOCX]

Movie Captions:

1. T_motion; monocular; sm
2. T_motion; synoptic; sm
3. T_motion; synoptic; dsm
4. T_motion; synoptic; mimo
5. T_motion; stereo; sm
6. T_motion; stereo; dsm
7. T_motion; stereo; mimo
8. RT_motion; synoptic; sm
9. RT_motion; synoptic; dsm
10. RT_motion; synoptic; mimo
11. RT_motion; stereo; sm
12. RT_motion; stereo; dsm
13. RT_motion; stereo; mimo
